# A case of lipomatous pleomorphic adenoma in the parotid gland: a case report

**DOI:** 10.1186/1746-1596-4-16

**Published:** 2009-06-04

**Authors:** Takeshi Kondo

**Affiliations:** 1 Division of Pathology, Department of Pathology, Kobe University Graduate School of Medicine, 7-5-1 Kusunoki-cho, Chuo-ku, Kobe 650-0017, Japan

## Abstract

**Introduction:**

Pleomorphic adenoma is the most common benign neoplasm of the salivary glands. Extensive lipomatous involvement of the tumor is, however, a very rare finding.

**Case report:**

Herein, a rare case of lipomatous pleomorphic adenoma arising in the parotid gland of a 14-year-old Japanese woman is presented.

**Conclusion:**

This is the sixth case of lipomatous pleomorphic adenoma in the English literature. Recognition of this rare subtype of pleomorphic adenoma is important for clinical diagnosis and management. On CT scan, it may not be detected possibly due to the extensive fatty component.

## Background

Pleomorphic adenoma is the most common benign neoplasm of the salivary glands, with the highest predilection for the parotid gland. The term 'pleomorphic' refers to both histogenesis and histology of the tumor. As the term 'pleomorphic' shows, the tumor is characteristic of the diversity of the histology. Lipomatous component can be included in pleomorphic adenoma. Extensive lipomatous involvement of the tumor is, however, a very rare finding in pleomorphic adenoma, with only five cases in the English literature [1-5]. Herein, I present the pathological findings of lipomatous pleomorphic adenoma.

## Case presentation

**Figure 1:**
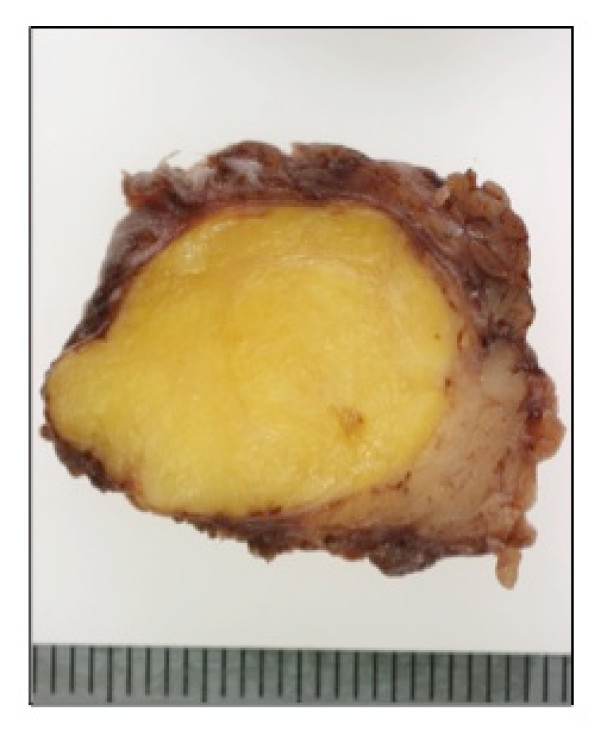
**Gross finding of the tumor (cut surface)**. Approximately 2 cm sized well-demarcated tumor of yellowish cut surface resembling lipoma.

**Figure 2:**
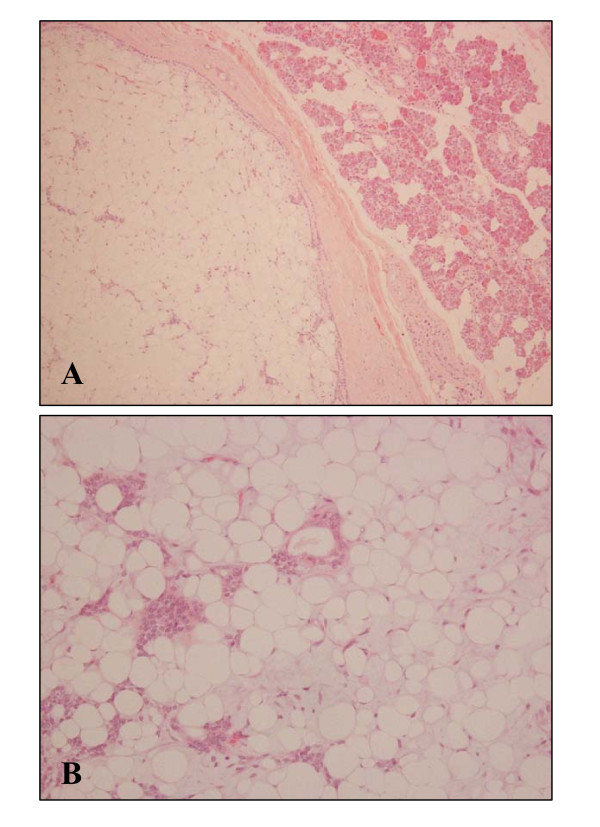
**Microscopic finding of the tumor**. A: well-demarcated lesion with fibrous capsule. Normal parotid gland can be seen (upper right). B: More than 95% of the tumor was adipose component, containing only scant epithelial components. In adipose tissue, there exists slight myxoid change (HE staining, A: ×100, B: ×200).

**Figure 3:**
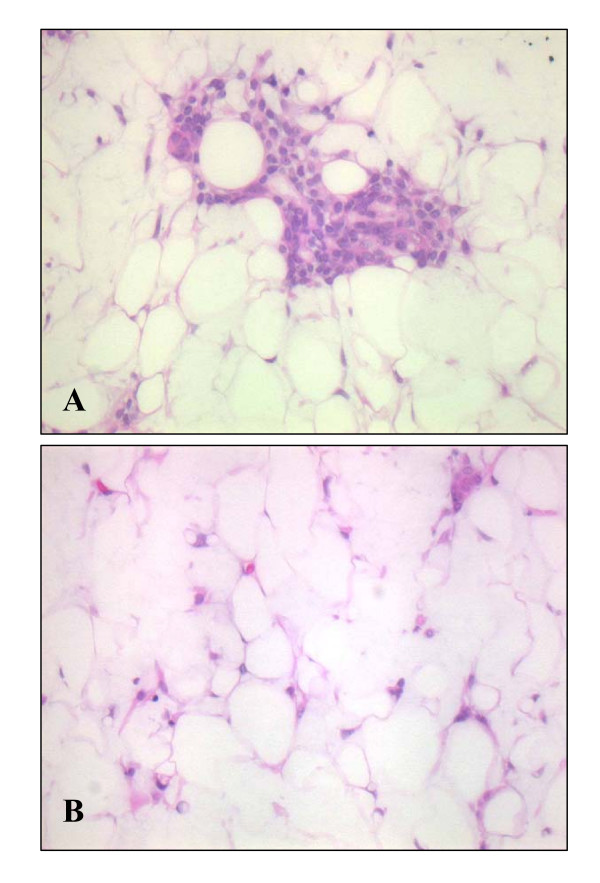
**Microscopic finding of the tumor (higher magnification)**. AB: More than 95% of the tumor was adipose component, containing only scant epithelial components. (HE staining, AB: ×400).

A 14-year-old Japanese woman presented with an asymptomatic slowly growing mass in the right preauricular region. CT scan was not performed. The patient then underwent excision of the deep lobe of the parotid gland. Grossly, the smooth surfaced lipomatous mass measured approximately 2 cm and had a uniform yellow cut surface (Fig. 1). Microscopic examination revealed a thick fibrous capsule completely surrounding the lesion (Fig. 2A). The well-encapsulated tumor consisted predominantly of mature adipose tissue (more than 95% of the entire lesion) with slight myxoid change containing only a scant epithelial element arranged in islands (Fig. 2B). Histologic images of higher magnification are shown in Fig. 3. Nuclear atypia or mitotic activity of epithelial cells was not identified. Adipocytes were univacuolar in shape, and no lipoblasts or malignant findings were identified. The tumor cells were negative for several special stains including PAS reaction (not shown). Although additional immunostaining was not performed for this case, these findings were diagnostic of a lipomatous pleomorphic adenoma. No recurrence has been experienced after surgery.

## Discussion

The term, lipomatous pleomorphic adenoma, was coined by Seifert et al. [1] in 1999, who defined this tumor as an otherwise typical pleomorphic adenoma with an abundant adipose component of more than 90% of the tumor area. This newly described variant is extremely rare and only five cases exist in the literature [1-5]. And this is the sixth case in the English literature. Additional file 1 summarizes the reported cases. No common symptoms of lipomatous pleomorphic adenoma were found.

Various forms of lipomatous tissue associations within salivary glands have been defined (lipoma, interstitial lipomatosis, lipoadenoma, oncocytic lipoadenoma, sialolipoma, and lipomatous atrophy). Although foci of adipose tissue are sometimes encountered within the stroma of pleomorphic adenoma, extensive replacement by adipose tissue like this case is a very rare finding. Differential diagnosis especially concerning lipoma (pure lipomatous tumor) is essential. The existence of epithelial or myoepithelial component is a diagnostic clue.

The histogenesis of lipomatous pleomorphic adenoma is not clear. Metaplastic transformation of myoepithelial cells to adipocytes and entrapment of fat tissue are two possible mechanisms.

Recognition of this rare form of pleomorphic adenoma is also important for clinical diagnosis and management. On CT scan, it may not be detected possibly due to the extensive fatty component that blends with the normal parotid gland.

## Conclusion

In conclusion, a rare case of lipomatous pleomorphic adenoma is presented, which is the sixth case in the English literature. Recognition of this rare subtype of is essential for clinical diagnosis, management, and treatment.

## Consent

Written informed consent was obtained from the patient for publication of this case report and accompanying images. A copy of the written consent is available for review by the Editor-in-Chief of this journal.

## Competing interests

The author declares that they have no competing interests.

## Authors' contributions

TK performed histological examination, analyzed the case, and wrote the manuscript.

## Supplementary Material

Additional File 1 **Table 1**. Summary of the reported case of lipomatous pleomorphic adenoma. Click here for file
