# Supplementary material for: A case of lipomatous pleomorphic adenoma in the parotid gland: a case report
Source: Diagn Pathol. 2009 Jun 4;4:16. doi: 10.1186/1746-1596-4-16 (PMC2710317; doi:10.1186/1746-1596-4-16)
Supplement: Additional File 1 — Table 1. Summary of the reported case of lipomatous pleomorphic adenoma. [file 1746-1596-4-16-S1.ppt]

## Slide 1
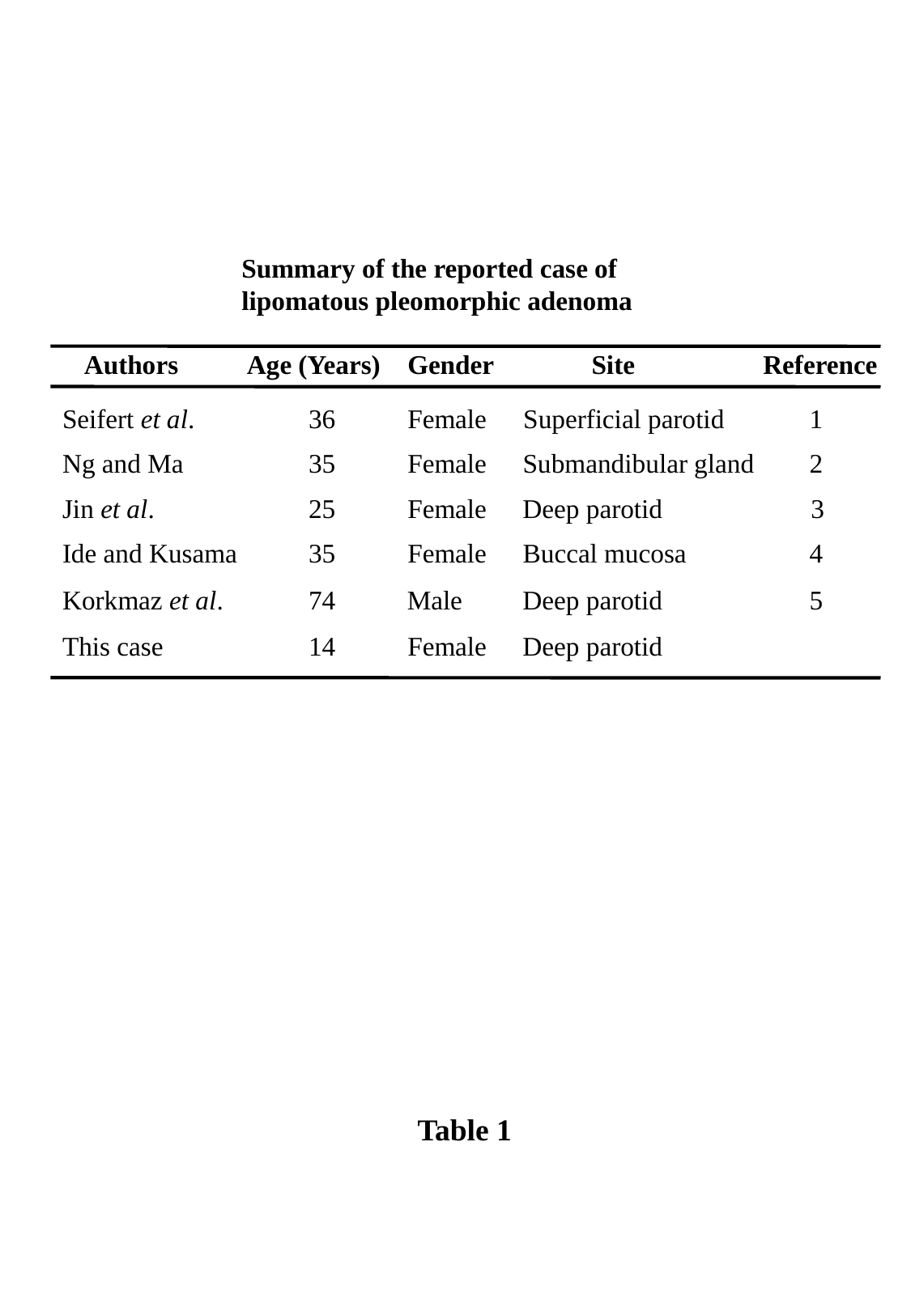

Summary of the reported case of
lipomatous pleomorphic adenoma
Authors
Age (Years)
Gender
Site
Reference
Seifert et al.
36
Female
Superficial parotid
1
Ng and Ma
35
Female
Submandibular gland
2
Jin et al.
25
Female
Deep parotid
3
Ide and Kusama
35
Female
Buccal mucosa
4
Korkmaz et al.
74
Male
Deep parotid
5
This case
14
Female
Deep parotid
Table 1
